# Location-Specific Radiomics Score: Novel Imaging Marker for Predicting Poor Outcome of Deep and Lobar Spontaneous Intracerebral Hemorrhage

**DOI:** 10.3389/fnins.2021.766228

**Published:** 2021-11-25

**Authors:** Zhiming Zhou, Hongli Zhou, Zuhua Song, Yuanyuan Chen, Dajing Guo, Jinhua Cai

**Affiliations:** ^1^Department of Radiology, Second Affiliated Hospital, Chongqing Medical University, Chongqing, China; ^2^Department of Radiology, Children‘s Hospital of Chongqing Medical University, Chongqing, China; ^3^Nanchong Central Hospital, Nanchong, China; ^4^Ministry of Education Key Laboratory of Child Development and Disorders, Children’s Hospital of Chongqing Medical University, Chongqing, China; ^5^Chongqing International Science and Technology Cooperation Center for Child Development and Disorders, Chongqing, China

**Keywords:** intracerebral hemorrhage, prognosis, radiomics, computed tomography, location

## Abstract

**Objective:** To derive and validate a location-specific radiomics score (Rad-score) based on noncontrast computed tomography for predicting poor deep and lobar spontaneous intracerebral hemorrhage (SICH) outcome.

**Methods:** In total, 494 SICH patients from multiple centers were retrospectively reviewed. Poor outcome was considered mRS 3–6 at 6 months. The Rad-score was derived using optimal radiomics features. The optimal location-specific Rad-score cut-offs for poor deep and lobar SICH outcomes were identified using receiver operating characteristic curve analysis. Univariable and multivariable analyses were used to determine independent poor outcome predictors. The combined models for deep and lobar SICH were constructed using independent predictors of poor outcomes, including dichotomized Rad-score in the derivation cohort, which was validated in the validation cohort.

**Results:** Of 494 SICH patients, 392 (79%) had deep SICH, and 373 (76%) had poor outcomes. The Glasgow Coma Scale score, haematoma enlargement, haematoma location, haematoma volume and Rad-score were independent predictors of poor outcomes (all *P* < 0.05). Cut-offs of Rad-score, 82.90 (AUC = 0.794) in deep SICH and 80.77 (AUC = 0.823) in lobar SICH, were identified for predicting poor outcomes. For deep SICH, the AUCs of the combined model were 0.856 and 0.831 in the derivation and validation cohorts, respectively. For lobar SICH, the combined model AUCs were 0.866 and 0.843 in the derivation and validation cohorts, respectively.

**Conclusion:** Location-specific Rad-scores and combined models can identify subjects at high risk of poor deep and lobar SICH outcomes, which could improve clinical trial design by screening target patients.

## Introduction

Spontaneous intracerebral hemorrhage (SICH) remains a devastating disease without effective therapies, with only approximately 20% of patients achieving functional independence at 6 months (van Asch et al., 2010). Recent studies have shown that the prognosis of SICH varies with the location of the haematoma, and clinical trials on this disease have begun to develop novel SICH treatments to target various populations to maximize treatment benefits (Anderson et al., 2010; Fu et al., 2014; Sreekrishnan et al., 2016; Qureshi and Qureshi, 2018). To better make clinical decisions for these candidate therapies, it is essential to derive effective and reliable prognostic tools for the prognostic risk assessment of SICH.

Haematoma volume is generally accepted as an easy-to-use and useful imaging marker for poor prognosis of SICH (Broderick et al., 1993; LoPresti et al., 2014). In some clinical trials for efficacy evaluation, the haematoma volume cut-off has been used as an eligibility criterion for screening the target population (Anderson et al., 2010; Fu et al., 2014; Qureshi and Qureshi, 2018). Nevertheless, using only the haematoma volume for prognostic evaluation may be unreliable because early haematoma enlargement (HE) occurs in approximately 30% of SICH patients (Brouwers and Greenberg, 2013). Recently, the spot sign, as an imaging marker for identifying patients with ongoing bleeding, has been added to the eligibility criterion for screening the target population in a clinical trial of tranexamic acid treatment (Meretoja et al., 2020). In addition, several imaging markers based on noncontrast computed tomography (NCCT), such as the blend sign and black hole sign, have been found to be associated with poor outcomes, which seems to provide a promising approach for rapidly identifying SICH patients at high risk of poor outcomes (Morotti et al., 2020a; Zimmer et al., 2020).

Unlike traditional imaging markers, radiomics features provide quantitative data extracted from medical images to quantify the phenotypic characteristics of diseases, which could provide potentially valuable information that is invisible to the naked eye (Lambin et al., 2012). Normally, the radiomics score (Rad-score) is developed based on the optimal radiomics features, which may comprehensively reflect the nature of lesions. Recently, several studies have reported that the Rad-score shows better performance than traditional imaging markers in predicting HE or poor outcomes after SICH (Xie et al., 2019; Song et al., 2021a,b). However, these recent studies do not take into account the potential differences in Rad-scores among different haematoma locations, which may be helpful for decision-making regarding the individualized treatment of SICH. To the best of our knowledge, there has been no attempt to identify an informative location-specific Rad-score cut-off as an eligibility criterion for proof-of-concept studies.

Therefore, we conducted a 2-phase study to derive and validate a novel location-specific Rad-score based on NCCT images for predicting 6-month poor outcome of deep and lobar SICH: first, we assessed the prognostic value of the Rad-score for the deep and lobar SICH and identified the location-specific Rad-score cut-offs; second, we derived and validated location-specific predictive models for poor prognosis after deep and lobar SICH based on Rad-score.

## Materials and Methods

### Patients

This retrospective study was approved by the Institutional Ethics Committee of our institution (No. [2019] 19), and the requirement for written informed consent was waived.

**FIGURE 1:**
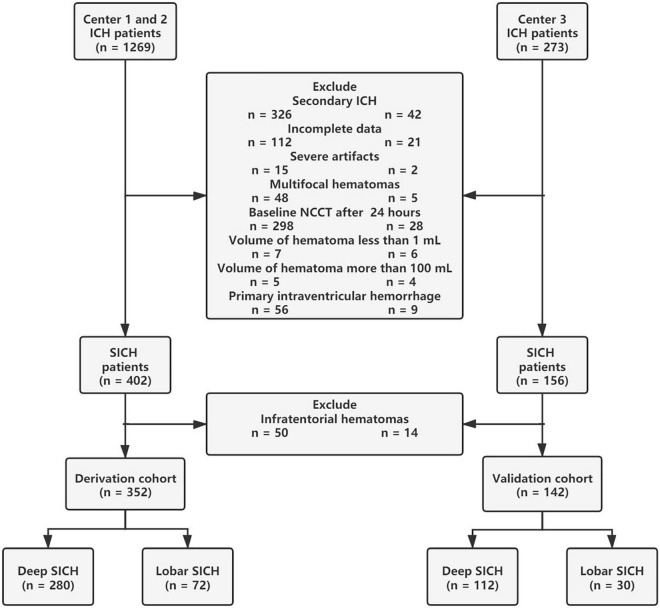
Flowchart of patient inclusion and exclusion criteria.

Between January 2014 and January 2021, a total of 1542 patients with intracerebral hemorrhage (ICH) from multiple centers were retrospectively enrolled in this study. ICH was diagnosed by head NCCT examination on admission. ICH patients over 18 years old were included in this study. The exclusion criteria for this study were (I) infratentorial haematomas; (II) multifocal haematomas; (III) haematoma volume <1 mL or >100 mL; (IV) ICH secondary to head trauma, vascular abnormality, haemorrhagic transformation of ischaemic stroke, or brain tumor; (V) primary intraventricular hemorrhage (IVH); (VI) baseline head NCCT examination over 24 h after symptom onset; (VII) severe artifacts on NCCT images; and (VIII) incomplete data. After patient screening, a total of 494 patients with SICH (352 patients in the derivation cohort and 142 patients in the validation cohort) were retrospectively reviewed in this study (Figure 1). All SICH patients were treated according to the hospital internal standard operating procedures and guidelines for the management of SICH (Morgenstern et al., 2010). Baseline characteristics, laboratory results, head NCCT examination, surgery interventions, HE and 6-month functional outcomes were collected for analysis. A known time from onset to baseline CT was recorded from the medical record system data, and the unknown onset-to-CT time was replaced by the median. In total, the onset-to-CT time of 11 (3%) patients in the derivation cohort and 5 (4%) patients in the validation cohort was unknown. Surgical interventions for SICH were divided into craniotomy and minimally invasive surgery, and the latter included endoscopic surgery and stereotactic aspiration.

### Outcome Assessment

The modified Rankin scale (mRS) at 6 months was assessed *via* standardized telephone interviews or medical records. Poor outcomes were defined as follows (Eriksson et al., 2007): the inability to walk outdoors or live in own home without assistance (mRS score = 3); the inability to walk or attend to own bodily needs without assistance (mRS score = 4); being bedridden, incontinent, and requiring constant nursing care and attention (mRS score = 5), or death (mRS score = 6). Good outcome was defined as mRS 0–2, whereas poor outcome was defined as mRS 3–6. HE was defined as >33% relative hemorrhage growth or >6 mL absolute hemorrhage growth on follow-up NCCT (Dowlatshahi et al., 2011).

### Image Acquisition and Analysis

Computed tomography (CT) examinations were performed on multislice spiral CT scanners following standard departmental protocols, with details described in the Supplementary Material. Image normalization, including image registration, gray level discretization, and a fixed head window, was performed before imaging analysis to reduce the possible influence of different scanners and scanning parameters, with details described in the Supplementary Material.

The haematoma locations were dichotomized into two groups: deep location involving the basal ganglia, thalamus, internal capsule, or deep periventricular white matter or lobar location involving the cortex and cortical-subcortical junction (Sembill et al., 2020). With reference to previous literature (Morotti et al., 2019), traditional imaging markers, including an irregular shape, black hole sign, blend sign and island sign, were independently assessed by individuals blinded to the outcome of SICH.

### Radiomics Analysis

First, haematoma segmentation was performed by manually delineating the region of interest (ROI) of SICH on the axial slices, and the haematoma volume was calculated automatically using ITK-SNAP software (version 3.8.0). Then, a total of 107 radiomics features were extracted automatically from each ROI using PyRadiomics software (version 3.0.1). All extracted radiomics features were from 7 feature classes: first-order statistics, shape-based features, grey level cooccurrence matrix (GLCM), grey level dependence matrix (GLDM), grey level run length matrix (GLRLM), grey level size zone matrix (GLSZM), and neighbouring grey tone difference matrix (NGTDM). A reproducibility analysis was performed to assess the stability of radiomics features, with details described in the Supplementary Material.

**FIGURE 2:**
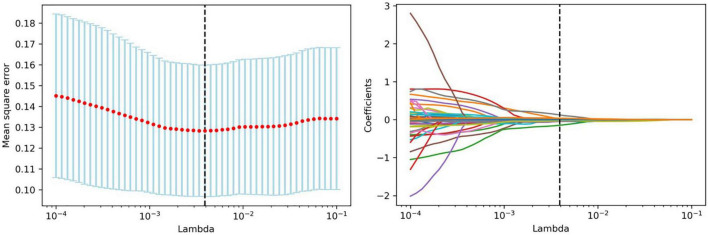
Feature selection with LASSO. Tuning parameter (lambda) selection in the LASSO model using 10-fold cross-validation. LASSO coefficient analysis of the 52 radiomics features. Each colored line represents the coefficient of each feature.

**FIGURE 3:**
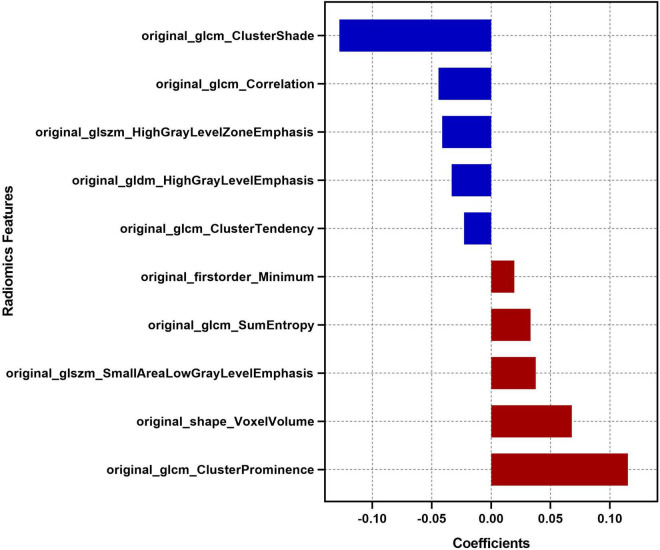
Descriptions of the 10 optimal radiomics features. The respective coefficient of each radiomics feature in the LASSO regression analysis is plotted on the *x*-axis.

Feature selection was performed using Python software (version 3.2). Before feature selection, harmonization in the feature domain was performed, with details described in the Supplementary Material. Then, univariable analysis was performed on 107 radiomics features, and 52 features with *P-*values <0.05 were retained. Then, least absolute shrinkage and selection operator (LASSO) regression was performed to select the optimized subset of features from the 52 features, with parameter tuning performed with 10-fold cross validation for overfitting diminution (Figure 2). Finally, only 10 optimal radiomics features were selected (Figure 3). Using these optimal features, the Rad-score was derived with the following formula: Rad-score $=(\sum_{1}^{i}\beta_{i}{\text{x}}_{i}$ +β_0_)× 100, where x_*i*_ is the i*^th^* selected radiomics feature, β_*i*_ is the respective coefficient determined by LASSO regression, and β_0_=0.832.

Receiver operating characteristic (ROC) curve analysis was performed, and the area under the ROC curve (AUC), sensitivity and a specificity were calculated to evaluate the predictive performance of the Rad-score in the deep and lobar SICH cohorts in the derivation and validation cohorts, respectively. Cut-off points for deep-specific and lobar-specific Rad-scores were identified using Youden’s index (YI). Sensitivity analyses were performed to determine the location-specific Rad-score cut-offs after excluding patients who withdrew from treatment.

### Model Building and Evaluation

A univariable analysis was performed to screen clinical and radiological variables associated with poor outcomes in the derivation cohort. Subsequently, significant (*P* < 0.05) variables were inputted into a multivariable logistic regression analysis using the backward stepwise method to identify independent predictors for poor outcome.

Independent predictors (*P* < 0.05) excluding the Rad-score were included in a multivariable logistic regression to construct clinical-radiological models in the deep and lobar SICH cohorts in the derivation cohort. The location-specific clinical-radiological models were independently validated in the validation cohort.

The Rad-scores were converted into dichotomous variables by referring to the cut-off points of the deep SICH and lobar SICH. Then, the combined models were constructed and visualized by adding the dichotomized Rad-score to the clinical-radiological models in the derivation cohort. The location-specific combined models were independently validated in the validation cohort.

Receiver operating characteristic curve analysis was performed, and the AUC, sensitivity and a specificity were calculated to evaluate the predictive performance of each model in the deep and lobar SICH cohorts.

### Statistical Analysis

Statistical analyses were performed using SPSS Statistics (version 19.0). Variables are expressed as the means ± standard deviations, medians (interquartile ranges [IQRs]), or frequencies (percentages) when appropriate. Shapiro–Wilk tests were applied to check the normality of continuous variables. The intergroup differences were compared with the chi-squared test, two-sample *t*-test, or Mann–Whitney *U*-test when appropriate. A two-tailed *P* < 0.05 was considered statistically significant.

## Results

The baseline characteristics of SICH patients in the derivation and validation cohorts are shown in Supplementary Table 1. Of the 494 SICH patients included in this study, 392 (79%) had deep SICH, and 102 (21%) had lobar SICH (Supplementary Table 2). Compared with patients with lobar SICH, patients with deep SICH had a significantly higher prevalence of history of hypertension (*P* < 0.001), higher admission systolic blood pressure (*P* = 0.002), shorter onset-to-CT time (*P* < 0.001), smaller haematoma volumes (*P* < 0.001), lower prevalence of irregular shape (*P* = 0.038) and island sign (*P* = 0.034), more IVH extension (*P* < 0.001), less subarachnoid hemorrhage (SAH) extension (*P* < 0.001), higher Rad-scores (*P* < 0.001), and more poor outcomes (*P* < 0.001).

In the derivation cohort, 262 (74%) patients had poor outcomes (mRS >2) at 6 months. In the univariable analysis, diabetes mellitus (*P* = 0.044), onset-to-CT time (*P* = 0.002), Glasgow Coma Scale (GCS) score (*P* < 0.001), white blood cell count (*P* = 0.006), serum glucose (*P* < 0.001), haematoma location (*P* < 0.001), haematoma volume (*P* = 0.012), black hole sign (*P* = 0.001), blend sign (*P* = 0.001), midline shift (*P* = 0.033), HE (*P* < 0.001), and Rad-score (*P* < 0.001) were associated with poor outcomes at 6 months (Table 1).

**TABLE 1:** Univariable analysis for poor outcome in the derivation cohort.

Variables	Good outcomes (*n* = 90)	Bad outcomes (*n* = 262)	*P*-value
Age, y	60.44 ± 11.97	61.74 ± 13.58	0.422
Male	58 (64.4)	161 (61.5)	0.706
Hypertension	52 (57.8)	168 (64.1)	0.313
Diabetes mellitus	14 (15.6)	69 (26.3)	0.044
Admission SBP, mmHg	172.74 ± 23.75	176.03 ± 31.26	0.362
Admission DBP, mmHg	101.04 ± 18.03	100.62 ± 19.63	0.856
Onset-to-CT time, h	4.13 ± 3.12	3.00 ± 2.12	0.002
GCS score	9.00 [7.00–10.00]	14.00 [13.00–14.00]	< 0.001
Surgical intervention	15(16.7)	58 (22.1)	0.295
Craniotomy	6 (6.7)	18 (6.9)	0.547
Minimal invasive surgery	9 (10.0)	40 (15.3)	
WBC, 10^9^/L	8.16 ± 2.82	9.22 ± 3.76	0.006
Hemoglobin, g/L	137.66 ± 20.01	134.34 ± 20.24	0.180
Platelets,10^9^/L	187.64 ± 70.85	178.76 ± 68.10	0.292
APTT, s	35.14 ± 6.43	35.04 ± 5.98	0.493
INR	1.02 ± 0.13	1.04 ± 0.18	0.347
Fibrinogen, g/L	3.06 ± 0.84	3.14 ± 1.04	0.421
Serum glucose, mmol/L	6.50 ± 1.80	7.49 ± 2.98	< 0.001
Hematoma location			< 0.001
Deep location	58 (64.4)	222 (84.7)	
Lobar location	32 (35.6)	40 (15.3)	
Hematoma volume, mL	14.41 ± 11.46	18.52 ± 14.01	0.012
1–29.9 mL	77 (85.6)	219 (83.6)	
30–59.9 mL	13 (14.4)	38 (14.5)	
60–100 mL	0 (0)	5 (1.9)	
Irregular shape	53 (58.9)	169 (64.5)	0.376
Black hole sign	9 (10.0)	68 (26.0)	0.001
Blend sign	6 (6.7)	56 (21.4)	0.001
Island sign	17 (18.9)	45 (17.2)	0.749
Midline shift	26 (28.9)	109 (41.6)	0.033
IVH	20 (22.2)	78 (29.8)	0.176
SAH	5 (5.6)	15 (5.7)	1.000
Rad-score	76.00 ± 8.11	86.49 ± 8.98	< 0.001
HE	15 (16.7)	125 (47.7)	< 0.001
6-month mRS	4.00 [3.00–4.00]	3.00 [2.00–4.00]	< 0.001

*Data are noted as means ± standard deviation, median and interquartile ranges or numbers and percentages in parenthesis. SBP, systolic blood pressure; DBP, diastolic blood pressure; CT, computed tomography; GCS, Glasgow Coma Scale; WBC, white blood cell; APTT, activated partial thromboplastin time; INR, international normalized ratio; IVH, intraventricular hemorrhage; SAH, subarachnoid hemorrhage; Rad-score, radiomics score; HE, haematoma enlargement; and mRS, modified Rankin Scale.*

In the multivariable analysis, GCS score (odds ratio [OR], 0.81; 95% confidence interval [CI], 0.69–0.95; *P* = 0.008), HE (OR, 0.42; 95% CI, 0.19–0.93; *P* = 0.033), haematoma location (OR, 0.31; 95% CI, 1.14–0.66; *P* = 0.002), haematoma volume (OR, 1.04; 95% CI, 1.01–1.08; *P* = 0.011), and Rad-score (OR, 1.15; 95% CI, 1.10–1.20; *P* < 0.001) were identified as independent predictors of poor outcomes at 6 months (Table 2). In the model adjusting for GCS score, HE, haematoma volume and Rad-score, haematoma location (OR, 0.32; 95% CI, 0.15–0.66; *P* = 0.002) was also independently associated with poor outcomes at 6 months.

**TABLE 2:** Multivariable analysis for poor outcomes in the derivation cohort.

Variable	OR	95%CI	*P-*value
Diabetes mellitus	0.66	0.28–1.57	0.348
Onset-to-CT time	0.97	0.86–1.09	0.598
GCS score	0.81	0.69–0.95	0.008
WBC	1.03	0.93–1.15	0.531
Serum glucose	1.06	0.91–1.25	0.464
Haematoma location	0.31	1.14–0.66	0.002
Haematoma volume	1.04	1.01–1.08	0.011
Black hole sign	0.73	0.29–1.80	0.493
Blend sign	0.67	0.23–1.94	0.455
Midline shift	0.87	0.43–1.76	0.689
Rad-score	1.15	1.10–1.20	< 0.001
HE	0.42	0.19–0.93	0.033

*OR, odds ratio; CI, confidence interval; CT, computed tomography; GCS, Glasgow Coma Scale; WBC, white blood cell; HE, haematoma enlargement; SICH, spontaneous intracerebral hemorrhage; and Rad-score, radiomics score.*

**FIGURE 4:**
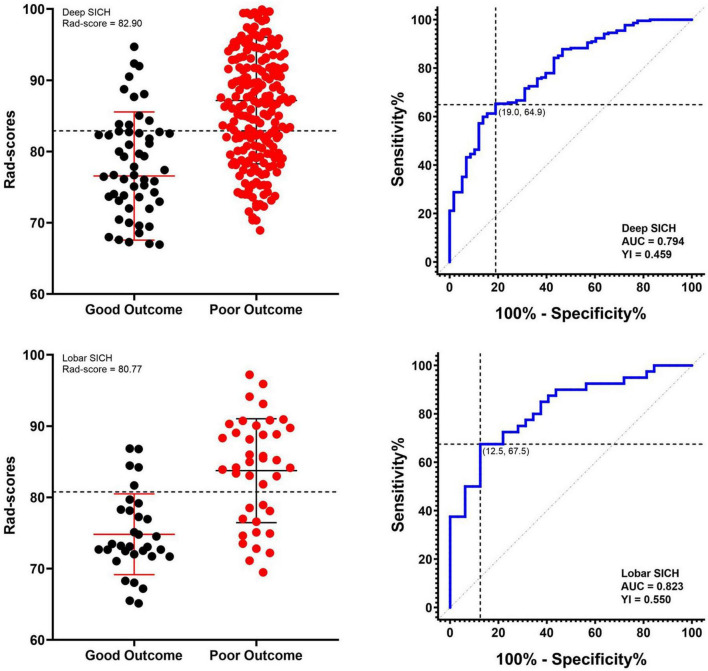
Receiver operating characteristic curves of the Rad-score and respective optimal cut-offs for predicting 6-month poor outcome in deep and lobar SICH cohorts in the derivation cohort.

In the derivation cohort, the Rad-score predicted poor outcomes with an AUC of 0.794, a sensitivity of 0.649 and a specificity of 0.810 in the deep SICH cohort and an AUC of 0.823, a sensitivity of 0.675 and a specificity of 0.875 in the lobar SICH cohort. ROC analysis identified a Rad-score of 82.90 (YI = 0.459) in the deep SICH and a Rad-score of 80.77 (YI = 0.550) in the lobar SICH as having optimal specificity and sensitivity for predicting poor outcomes at 6 months (Figure 4). Thus, deep SICH patients with Rad-scores >82.90 or lobar SICH patients with Rad-scores >80.77 were predicted to have poor outcomes. In the validation cohort, the Rad-score predicted poor outcomes with an AUC of 0.783, a sensitivity of 0.634 and a specificity of 0.789 in the deep SICH cohort and an AUC of 0.806, a sensitivity of 0.611 and a specificity of 0.833 in the lobar SICH cohort. After exclusion of patients who withdrew from treatment (*n* = 23), the location-specific Rad-score cut-off did not change significantly, with a Rad-score of 82.84 (YI = 0.454) in the deep SICH cohort and Rad-score of 80.77 (YI = 0.567) in the lobar SICH cohort.

**FIGURE 5:**
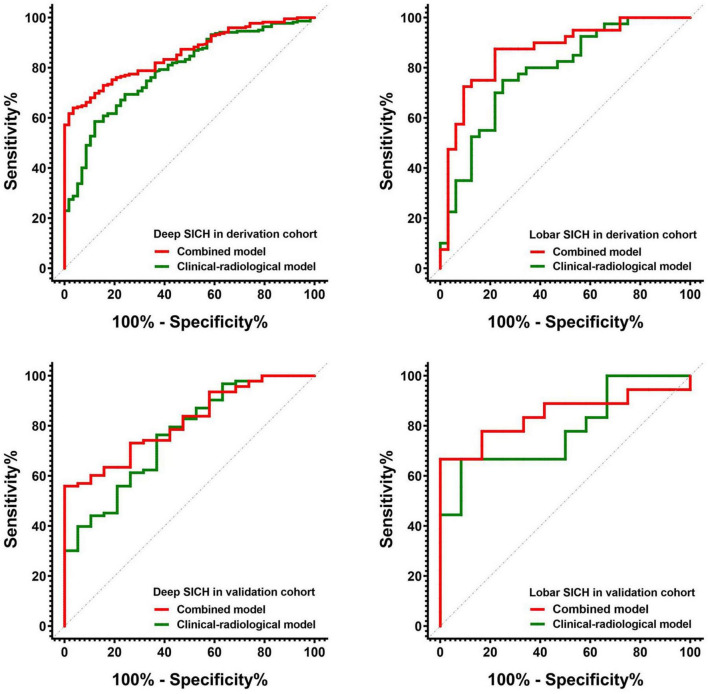
Receiver operating characteristic curves of the clinical-radiological models and combined models for predicting 6-month poor outcome in deep and lobar SICH cohorts from the derivation and validation cohorts.

**FIGURE 6:**
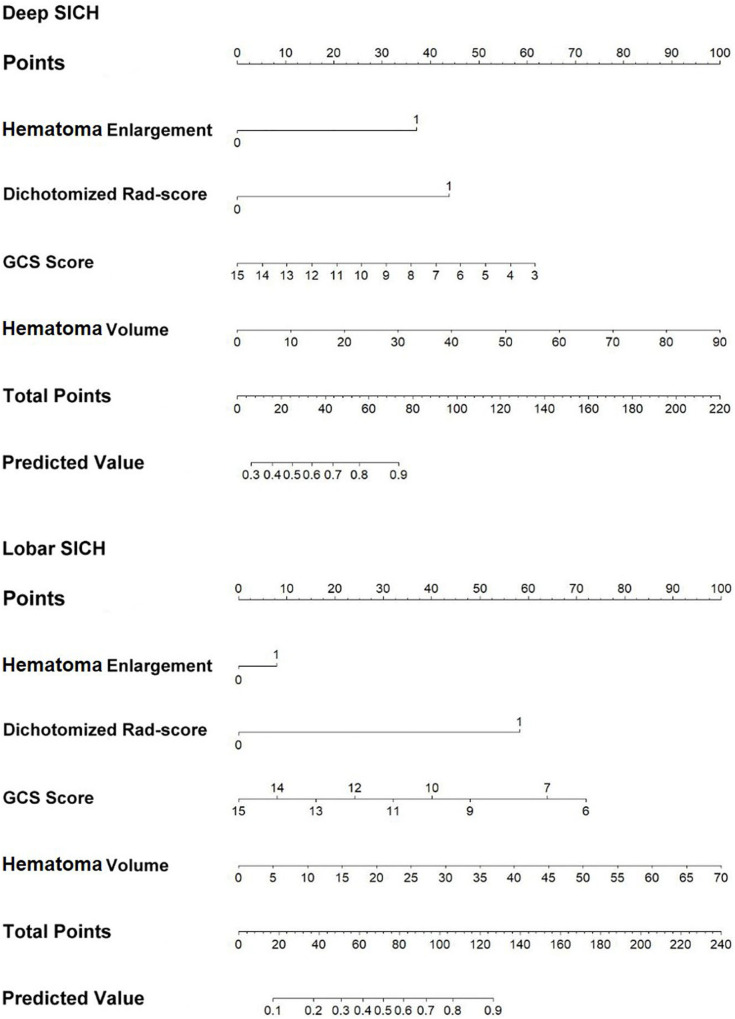
Nomograms of the location-specific combined models for predicting 6-month poor outcome of deep and lobar SICH.

In the derivation cohort, the clinical-radiological model including GCS score, HE, and haematoma volume predicted poor outcomes with an AUC of 0.790, a sensitivity of 0.784 and a specificity of 0.638 in the deep SICH cohort and an AUC of 0.783, a sensitivity of 0.625 and a specificity of 0.781 in the lobar SICH cohort (Figure 5). After including the dichotomized Rad-score, the location-specific combined model predicted poor outcomes with an AUC of 0.856, a sensitivity of 0.784 and a specificity of 0.776 in the deep SICH cohort and an AUC of 0.866, a sensitivity of 0.775 and a specificity of 0.781 in the lobar SICH cohort (Figure 5). In the validation cohort, the clinical-radiological model predicted poor outcomes with an AUC of 0.762, a sensitivity of 0.763 and a specificity of 0.632 in the deep SICH cohort and an AUC of 0.782, a sensitivity of 0.667 and a specificity of 0.750 in the lobar SICH cohort (Figure 5). The location-specific combined model predicted poor outcomes with an AUC of 0.831, a sensitivity of 0.720 and a specificity of 0.789 in the deep SICH cohort and an AUC of 0.843, a sensitivity of 0.778 and a specificity of 0.750 in the lobar SICH cohort (Figure 5). The nomograms of the location-specific combined models are shown in Figure 6.

## Discussion

To our knowledge, our study was a new attempt to derive an easy-to-use location-specific Rad-score to predict poor outcomes at 6 months. Using the location-specific Rad-score, we found that a Rad-score of 82.90 in the deep SICH and a Rad-score of 80.77 in the lobar SICH as the cut-offs with the maximum specificity and sensitivity for predicting poor outcomes in patients with SICH. Furthermore, we derived and validated two location-specific combined models with independent predictors, including the dichotomized Rad-score, to predict poor outcomes of deep and lobar SICH. The location-specific combined models achieved satisfactory prediction performances and could provide effective auxiliary tools for screening SICH patients at high risk of poor outcomes at 6 months.

Recently, most SICH trials and SICH treatment paradigms have used the same eligibility criteria to screen the target population from patients with deep or lobar SICH without attempting finer topographic comparisons (Sreekrishnan et al., 2016). Deep and lobar SICH have different risk factors and prognostic outcomes because they arise from distinct cerebral small vessel diseases (Pantoni, 2010). In this study, a higher prevalence of history of hypertension and higher systolic blood pressure were found in deep SICH patients, which may be associated with more hypertensive arteriopathy. In contrast, larger haematoma volumes and more SAH extension occurred in lobar SICH patients, which was consistent with the findings of a previous study (Morotti et al., 2020b). In addition, our results suggested that deep and lobar SICH need to be treated differently because their prognostic outcomes were significantly different. It is particularly worth mentioning here that there were significant differences in Rad-scores and poor outcomes between patients stratified by lobar and deep SICH location, which supported our hypothesis that location-specific Rad-scores may be helpful in the assessment of the risk of poor outcomes of SICH.

The Rad-score proposed in this study was a comprehensive manifestation of the characteristics of haematomas. The Rad-score we constructed was a model using 10 optimal radiomics features selected from 107 extracted radiomics features of haematoma images. Among the 10 optimal radiomics features, the “Minimum” feature reflects the minimum signal intensity of a haematoma; the “Voxel Volume” feature reflects the volume of a haematoma; the three “GLCM Cluster” features reflect the image gray level value and the gray level difference between the forms of haematomas; the “GLCM Entropy” feature reflects the neighborhood intensity value differences in haematoma images; the “GLCM Correlation” feature reflects the linear dependency of gray level values of haematoma images; the “GLDM” feature reflects the distribution of the higher gray level values of haematoma images; the two “GLSZM” features characterize the consistency of texture, aperiodic or speckle texture within the haematoma (Zwanenburg et al., 2020). Thus, the Rad-score contained multidimensional information about the heterogeneity of the haematoma and was determined to be a radiological independent predictor of poor outcomes in this study. In addition, the Rad-score can be obtained by only drawing the ROI of the haematoma and automatic computer calculation, which may reduce the dependence on imaging diagnosis experience and the influence of subjective factors (Ma et al., 2019). As a quantification and full extension of traditional imaging markers, the Rad-scores can quantitatively reflect the nature of haematomas comprehensively and objectively.

On the other hand, the location-specific Rad-score that took into account the potential interaction between Rad-score and haematoma location can help predict poor outcomes at 6 months. Most recently, several studies suggested that NCCT radiomics features of haematomas could improve the predictive performance of prognostic models (Pszczolkowski et al., 2021; Song et al., 2021b). Nevertheless, the differences in radiomics features by haematoma location were not considered in these models, which may be valuable for proof-of-concept studies (Anderson et al., 2010; Fu et al., 2014; Qureshi and Qureshi, 2018). Our results showed that both haematoma location and Rad-score were independent predictors of poor outcomes at 6 months. In the stratified analysis, the Rad-score achieved good performance in predicting poor outcomes, especially in the lobar SICH cohort. In addition, we identified a Rad-score of 82.90 in the deep SICH and a Rad-score of 80.77 in the lobar SICH as the optimal cut-offs for predicting poor outcomes in SICH patients. Recently, studies have performed much work on location-specific haematoma volume cut-offs suitable for proof-of-concept trials (Ironside et al., 2019; Leasure et al., 2019). Compared with the single feature of location-specific volume, our novel location-specific Rad-score threshold may be a more comprehensive and stable novel marker for predicting poor outcomes.

While a variety of prognostic models for SICH have been developed, most models do not account for differences associated with haematoma location (Gregorio et al., 2018). In view of the different pathologies and prognoses of SICH in different locations (Pantoni, 2010; Eslami et al., 2019), it is essential to derive novel location-specific models to predict poor outcomes. Taking into account the possible influence of haematoma locations on prediction models, we constructed and validated two combined models suitable for use in lobar and deep SICH by adding the dichotomized Rad-score to the clinical-radiological models. Our results showed that the location-specific combined models achieved the best predictive performances in both the derivation and validation cohorts. Therefore, our findings provide novel location-specific combined models with GCS score, HE, haematoma volume and dichotomized Rad-score to help effectively assess the risk of poor outcome among patients with deep or lobar SICH.

Several limitations deserve consideration in this study. First, this study was limited by its retrospective nature, relatively small sample size and unbalanced dataset, and the clinical pathway was not extremely strict. Prospective validation is required, and additional optimization of the location-specific Rad-score cut-offs and combined models may be necessary. Second, the baseline haematoma volumes of patients were relatively small, which may have an impact on the evaluation of the relationship between haematoma volumes and poor outcomes. Third, the generalizability of all models in this study may be limited due to the inclusion and exclusion of patients.

## Conclusion

Our results indicated the good prognostic value of the novel location-specific Rad-score for deep and lobar SICH. More importantly, we provided location-specific Rad-score cut-offs and combined models for predicting poor outcomes of patients with deep and lobar SICH at 6 months. These novel location-specific markers and combined models could help assess the risk of poor prognosis and improve clinical trial design.

## Data Availability Statement

The original contributions presented in the study are included in the article/Supplementary Material, further inquiries can be directed to the corresponding author.

## Ethics Statement

The studies involving human participants were reviewed and approved by the Institutional Ethics Committee of the Second Affiliated Hospital of Chongqing Medical University. Written informed consent for participation was not required for this study in accordance with the national legislation and the institutional requirements.

## Author Contributions

JC and ZZ: conception, design, analysis, and interpretation of data. HZ, YC, ZS, and ZZ: acquisition of data. YC and ZZ: data processing and statistical analysis. JC and ZZ: writing the first draft of the manuscript. DG, JC, and ZZ: critical revision of manuscript for intellectual content and study supervision. DG and JC: technical and administrative supports. JC and ZZ: approving the final version of the manuscript on behalf of all authors. All authors contributed to the article and approved the submitted version.

## Conflict of Interest

The authors declare that the research was conducted in the absence of any commercial or financial relationships that could be construed as a potential conflict of interest.

The reviewer ZG has declared a shared parent affiliation with the authors ZZ, ZS, YC, DG, and JC at the time of review.

## Publisher’s Note

All claims expressed in this article are solely those of the authors and do not necessarily represent those of their affiliated organizations, or those of the publisher, the editors and the reviewers. Any product that may be evaluated in this article, or claim that may be made by its manufacturer, is not guaranteed or endorsed by the publisher.
