# Corrigendum: Location-Specific Radiomics Score: Novel Imaging Marker for Predicting Poor Outcome of Deep and Lobar Spontaneous Intracerebral Hemorrhage

**DOI:** 10.3389/fnins.2022.829130

**Published:** 2022-03-11

**Authors:** Zhiming Zhou, Hongli Zhou, Zuhua Song, Yuanyuan Chen, Dajing Guo, Jinhua Cai

**Affiliations:** ^1^Department of Radiology, Second Affiliated Hospital, Chongqing Medical University, Chongqing, China; ^2^Department of Radiology, Children‘s Hospital of Chongqing Medical University, Chongqing, China; ^3^Nanchong Central Hospital, Nanchong, China; ^4^Ministry of Education Key Laboratory of Child Development and Disorders, Children's Hospital of Chongqing Medical University, Chongqing, China; ^5^Chongqing International Science and Technology Cooperation Center for Child Development and Disorders, Chongqing, China

**Keywords:** intracerebral hemorrhage, prognosis, radiomics, computed tomography, location

In the original article, there was a mistake in Figure 6 as published. The value of the slide rule for the hematoma enlargement in the nomogram of the lobar SICH was reversed. The corrected Figure 6 appears below.

**Figure 6:**
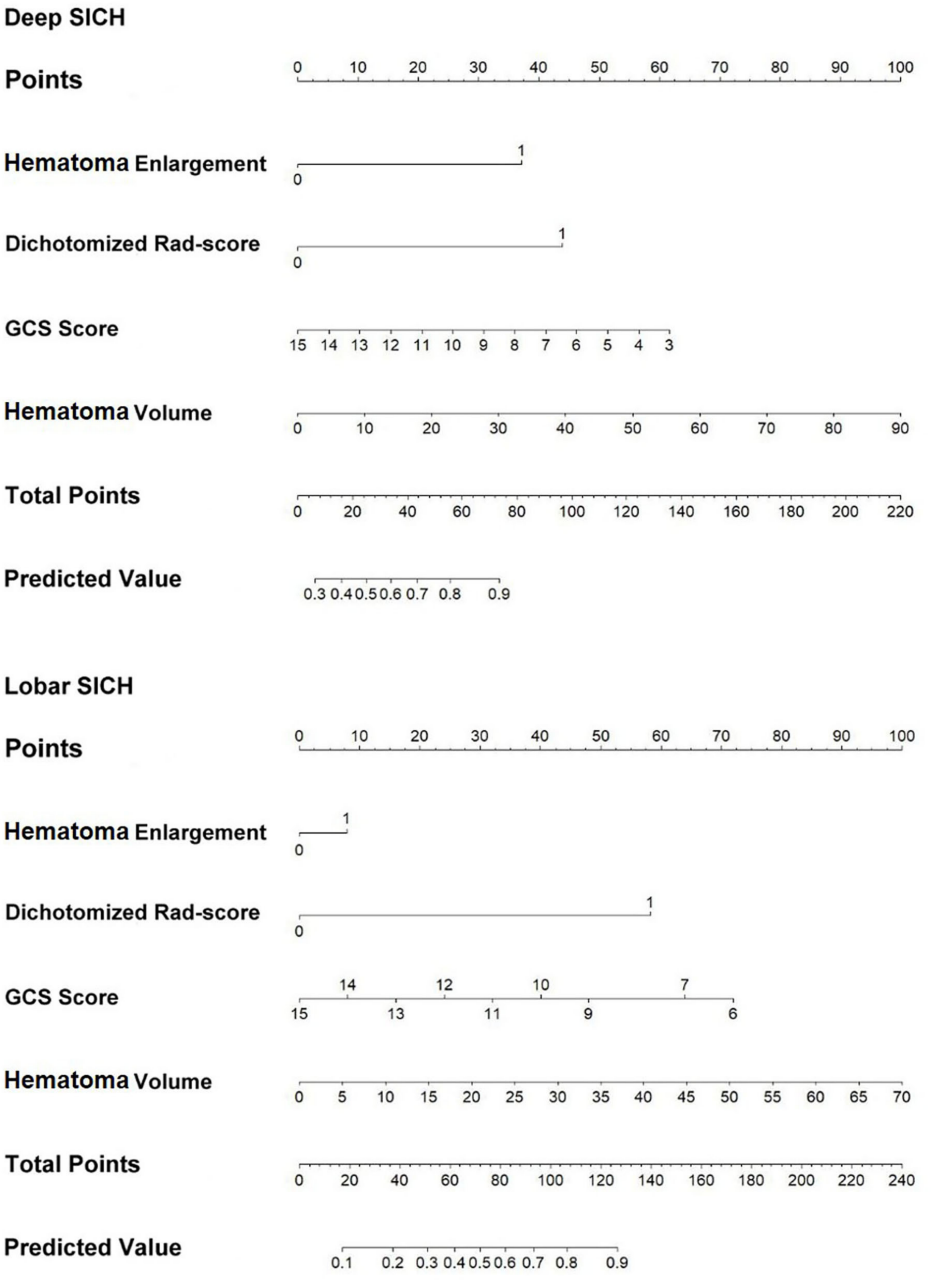
Nomograms of the location-specific combined models for predicting 6-month poor outcome of deep and lobar SICH.

The authors apologize for this error and state that this does not change the scientific conclusions of the article in any way. The original article has been updated.

## Publisher's Note

All claims expressed in this article are solely those of the authors and do not necessarily represent those of their affiliated organizations, or those of the publisher, the editors and the reviewers. Any product that may be evaluated in this article, or claim that may be made by its manufacturer, is not guaranteed or endorsed by the publisher.

