# Supplementary material for: Location-Specific Radiomics Score: Novel Imaging Marker for Predicting Poor Outcome of Deep and Lobar Spontaneous Intracerebral Hemorrhage
Source: Front Neurosci. 2021 Nov 25;15:766228. doi: 10.3389/fnins.2021.766228 (PMC8656420; doi:10.3389/fnins.2021.766228)
Supplement: Supplementary file 1 [file Data_Sheet_1.pdf]

Supplemental Materials

MATERIALS AND METHODS

Imaging acquisition

All NCCT scans were performed by 3 different CT scanners (Ingenuity, Philips Healthcare; Somatom Force, Siemens Healthcare; Aquilion ONE, Canon medical Systems) using the following scanning parameters: a tube voltage of 120 kV, 250-300 mA, matrix size of 512 × 512, gantry rotation of 0.4-0.6 s, field of view of 25 cm and slice thickness of 1 mm or 5 mm, and a detector width of 0.625 mm or 0.5 mm. The scanning range was from the skull base to the cranium.

Image normalization

Image normalization was performed as follows: (a) image registration: every noncontrast computed tomography (NCCT) image slice from the raw data was resampled to a unified pixel dimension size of $1.0 \times 1.0 \times 1.0$ mm^3; (b) gray level discretization: image intensity of every NCCT image were normalized by the gray level discretization method with a fixed number of bins (256 bins); (c) NCCT images were viewed in a fixed head window (level = 50 Hounsfield unit; width = 110 Hounsfield unit).

Radiomics analysis

Reproducibility analysis

Inter-observer and intra-observer reproducibility analyses were performed on 50 patients randomly selected from derivation cohort. The region of interest (ROI) of each patient was manually segmented again by the same radiologist after 2 weeks interval and by another radiologist using the same method. After extracting radiomics features, the intraclass correlation coefficient (ICC) was calculated to assess the reproducibility of radiomics features. The results showed that 98 (91.6%) radiomics features showed ICC > 0.8 in the inter-observer reproducibility analysis and 100 (93.5%) radiomics features showed ICC > 0.8 in the intra-observer reproducibility analysis.

Radiomics Features Harmonization

Before radiomics features selection, harmonization in the feature domain was performed. First, variables with zero variance were excluded from analyses, and the missing values were replaced by the median. Then the data were standardized by using zero-mean normalization.

**Table S1 | ** Patients' characteristics in the derivation and validation cohorts.

Variables	Derivation cohort (n = 352)	Validation cohort (n = 142)	*P* value
Age, y	61.41 ± 13.18	60.89 ± 10.12	0.636
Male	219 (62.2)	86 (60.6)	0.759
Hypertension	220 (62.5)	88 (62)	0..919
Diabetes mellitus	83 (23.6)	33 (23.2)	1.000
Admission SBP, mmHg	175.19 ± 29.52	171.62 ± 26.32	0.210
Admission DBP, mmHg	100.73 ± 19.21	99.11 ± 16.77	0.381
Onset-to-CT time, h	3.28 ± 2.46	3.07 ± 1.62	0.324
GCS	13.00 [10.00-14.00]	13.00 [11.00-14.00]	0.485
Surgical intervention	73 (20.7)	28 (19.7)	0.902
Craniotomy	24 (6.8)	8 (5.6)	0.812
Minimal invasive surgery	49 (13.9)	20 (14.1)	
WBC, 10^9/L	8.95 ± 3.57	8.67 ± 3.49	0.428
Hemoglobin, g/L	135.19 ± 20.21	135.84 ± 16.48	0.734
Platelets,10^9/L	181.03 ± 68.82	177.29 ± 60.71	0.572
APTT, s	34.30 ± 6.22	34.73 ± 5.67	0.462
INR	1.04 ± 0.17	1.04 ± 0.21	0.736
Fibrinogen, g/L	3.07 ± 1.00	3.12 ± 0.95	0.616
Serum glucose, mmol/L	7.24 ± 2.76	7.04 ± 2.51	0.457
Hematoma location			0.902
Deep location	280 (79.5)	112 (78.9)	
Lobar location	72 (20.5)	30 (21.1)	
Hematoma volume, mL	17.48 ± 13.51	18.86 ± 11.29	0.281
1-29.9 mL	296 (84.1)	122 (85.9)	
30-59.9 mL	51 (14.5)	19 (13.4)	
60-100 mL	5 (1.4)	1 (0.7)	
Irregular shape	222 (63.1)	91 (64.1)	0.918
Black hole sign	77 (21.9)	35 (24.6)	0.553
Blend sign	62 (17.6)	24 (16.9)	0.896
Island sign	62 (17.6)	18 (12.7)	0.224
Midline shift	135 (38.4)	46 (32.4)	0.218
IVH	98 (27.8)	40 (28.2)	1.000
SAH	20 (5.7)	8 (5.6)	1.000
Rad-score	83.81 ± 9.88	84.14 ± 8.07	0.699
HE	140 (39.8)	52 (36.6)	0.542
6-month mRS	2.00 [2.00-2.00]	3.00 [3.00-4.00]	0.647
Poor outcome	262 (74.4)	111 (78.2)	0.420

Data are noted as means ± standard deviation, median and interquartile ranges or numbers and percentages in parenthesis. SBP, systolic blood pressure; DBP, diastolic blood pressure; CT, computed tomography; GCS, Glasgow Coma Scale; WBC, white blood cell; APTT, activated partial thromboplastin time; INR, international normalized ratio; IVH, intraventricular hemorrhage; SAH,

subarachnoid hemorrhage; Rad-score, radiomics score; HE, hematoma enlargement; and mRS, modified Rankin Scale.

Table S2 | Comparison of baseline characteristics between patients with deep and lobar SICH.

**Variables**	**Lobar SICH (n = 102)**	**Deep SICH (n = 392)**	***P* value**
Age, y	62.40 ± 12.81	60.96 ± 12.25	0.295
Male	71 (69.6)	234 (59.7)	0.068
Hypertension	40 (39.2)	268 (68.4)	< 0.001
Diabetes mellitus	28 (27.5)	88 (22.4)	0.296
Admission SBP, mmHg	166.28 ± 28.60	176.22 ± 28.35	0.002
Admission DBP, mmHg	98.24 ± 22.95	100.79 ± 17.20	0.295
Onset-to-CT time, h	4.06 ± 2.63	3.01 ± 2.10	< 0.001
GCS	13.00 [11.00-14.00]	13.00 [10.00-14.00]	0.574
Surgical intervention	25 (24.5)	76 (19.4)	0.271
Craniotomy	11 (10.8)	21 (5.4)	0.143
Minimal invasive surgery	14 (13.7)	55 (14.0)	
WBC, 10^9/L	8.89 ± 3.91	8.86 ± 3.45	0.950
Hemoglobin, g/L	133.73 ± 16.18	135.80 ± 19.90	0.331
Platelets,10^9/L	181.30 ± 73.77	179.61 ± 64.63	0.819
APTT, s	34.07 ± 6.57	34.52 ± 5.93	0.510
INR	1.08 ± 0.31	1.03 ± 0.13	0.138
Fibrinogen, g/L	3.14 ± 1.05	3.07 ± 0.97	0.479
Serum glucose, mmol/L	6.92 ± 1.87	7.25 ± 2.86	0.155
Hematoma volume, mL	21.66 ± 13.11	16.56 ± 12.72	< 0.001
1-29.9 mL	75 (73.5)	343 (87.5)	
30-59.9 mL	25 (24.5)	45 (11.5)	
60-100 mL	2 (2.0)	4 (1.0)	
Irregular shape	74 (72.5)	239 (61.0)	0.038
Black hole sign	28 (27.5)	84 (21.4)	0.232
Blend sign	15 (14.7)	71 (18.1)	0.466
Island sign	24 (23.5)	56 (14.3)	0.034
Midline shift	42 (41.2)	139 (35.5)	0.300
IVH	13 (12.7)	125 (31.9)	< 0.001
SAH	15 (14.7)	13 (3.3)	< 0.001
Rad-score	80.16 ± 8.39	84.91 ± 9.66	< 0.001
HE	46 (45.1)	146 (37.2)	0.171
6-month mRS	3.00 [2.00-4.00]	3.00 [3.00-4.00]	0.022
Poor outcome	58 (56.9)	315 (80.4)	< 0.001

Data are noted as means ± standard deviation, median and interquartile ranges or numbers and percentages in parenthesis. SICH, spontaneous intracerebral hemorrhage; SBP, systolic blood pressure; DBP, diastolic blood pressure; CT, computed tomography; GCS, Glasgow Coma Scale; WBC, white blood cell; APTT, activated partial thromboplastin time; INR, international normalized ratio; IVH, intraventricular hemorrhage; SAH, subarachnoid hemorrhage; Rad-score, radiomics score; HE, hematoma enlargement; and mRS, modified Rankin Scale.
